# Optoelectronic refractometric sensing device for gases based on dielectric bow-ties and amorphous silicon solar cells

**DOI:** 10.1038/s41598-022-21299-w

**Published:** 2022-11-01

**Authors:** Mahmoud H. Elshorbagy, Óscar Esteban, Alexander Cuadrado, Javier Alda

**Affiliations:** 1 Physics Department, Faculty of Science, Minia University, 61519 El-Minya, Egypt; 2 Faculty of Optics and Optometry, Applied Optics Complutense Group, University Complutense of Madrid, C/Arcos de Jalón, 118, 28037 Madrid, Spain; 3 Photonics Engineering Group, University of Alcalá, 28801 Alcalá de Henares, Madrid Spain; 4 Escuela de Ciencias Experimentales y Tecnología, University Rey Juan Carlos, 28933 Móstoles, Madrid, Spain

**Keywords:** Nanoscale devices, Nanoscience and technology, Optics and photonics

## Abstract

The transformation of an hydrogenated amorphous silicon solar cell (aSiH) into an optoelectronic refratometric sensor has been possible through the addition of dielectric bow-tie resonant structures. The indium transparent oxide top electrode is replaced by a thin metallic layer to selectively prevent the direct transmission of light to the active layer of the cell. Then, an array of dielectric bow-tie structures is placed on top of this electrode, to activate the optical absorption through surface plasmon resonance (SPR). The whole device is exposed to the analyte under measure, which is the surrounding medium. Three different dielectric materials with low, medium, and high refractive index were selected for the bow-ties, namely magnesium fluoride (MgF$_2$), silicon dioxide (SiO$_2$), and aluminum nitride (AlN) have been tested as coupling structure for SPR excitation. The maximization of the readout/short circuit current has been achieved through the geometrical parameters of such structure. We have selected the geometrical parameters to maximize the short circuit current delivered by the a-Si cell at a given selected wavelength. The design has been customized to gas measurements application, where the index of refraction is slightly above 1 around 10$^{-4}$. Our analysis reveals ultra-high sensitivity of $2.4 \times 10^4$ (mA/W)/RIU, and a figure of merit FOM= 107 RIU$^{-1}$, when the bow-tie is made of SiO$_2$. A performance rally competitive with those previously reported in literature, with the additional advantage of circunventing both moving parts and spectral interrogation elements.

## Introduction

Optical sensing based on plasmonic effect has been applied in many areas over the last three decades^1,2^. This technology has been demonstrated for material identification^3^, food quality assurance^4^, colorimetry^5^, environmental quality tests^6^, or biosensing applications^7^. Focusing on environmental applications, high resolution and sensitivity devices are required to detect very small quantities of atmospheric pollutants like hydrocarbons, volatile organic compounds, microbiological hazards, etc.^8–14^. The monitoring of air quality and composition can be made through the measurement of its index of refraction^15,16^, that also depends on other physical parameters (temperature and pressure), and the chemical composition of the atmosphere (humidity, presence of natural or artificial specimens)^17^. As a consequence, a refractometric sensor can check if some preset conditions are fulfilled, or a known specimen varies its concentration.

Due to their narrow selective response, opto-electronic devices based on surface plasmon resonances (SPR) are one of the solutions for environmental monitoring and sensing^18,19^. This technology can be applied to gas sensing^20^, refractive index sensing^21–24^, and chemical sensing^25^. They can also be included in multi-functional and multiparametric sensors^26^. Plasmonic sensor can be interrogated angularly, spectrally, and opto-electronically. When using the angular dependence of the plasmonic response, the system usually requires moving parts and a high-precision goniometer or expensive integrated readout systems^27^. The same happens with the spectral interrogation: it needs high-resolution monochromators in the illumination and/or the detection arms^28^. The pure opto-electronic interrogation method benefits from the electric signal delivered by the sensor itself without needing moving parts and/or monochromators. This fact eases the interrogation subsystems and makes a more compact and more reliable sensor^29^.

A solar cell can be seen as an already-made and low-cost light detector. Although designed as a wide spectral photovoltaic detector, it can be easily transformed to respond selectively through the excitation of SPRs generated by nanostructured metasurfaces^30^. As a whole, the customized device becomes a self-powered optoelectronic sensor^20^. Organic and inorganic thin film solar cells are the best candidates for low-cost, light-weight and compact sensing devices^31^. Among them, hydrogenated amorphous silicon (aSiH) cells are commercial devices that employ an abundant, non-toxic and stable material^32,33^ at a quite affordable price. Then, aSiH will be considered in this paper as the base device that will be transformed to work as a refractometric gas sensor interrogated opto-electronically^34^.

In this contribution, we numerically analyze and quantitatively describe how to modify an aSiH cell to sense changes in controlled atmospheres. Although several optical technologies have been applied to sense refractometric changes in gases^15,16^, most of them are expensive and complex to operate. Our system is based on the selective opto-electronic response of a solar cell where its exposed surface is populated with dielectric bow-tie resonant structures sitting on a thin-film metallic front electrode that replace the classical transparent ITO layer.

## Material and methods

A simulation package COMSOL Multiphysics based on finite element method is used to solve Maxwell equations for the optical fields distribution inside every layer in the three dimensional model of the device. These fields are used to calculate absorption at each layer. The extracted current from the device is directly proportional to the absorption in the active layer of the device. Finally, we weight this current to the incident optical power to obtain the responsivity^18^. The modeling domain is a small unit cell or the building block of a large array, which is repeated transversely by applying periodic boundary conditions. An excitation port with a customized wavelength, intensity, and orientation is placed on top of the whole structure as illumination source. At the top of the illumination source we place a perfect matching layer (PML) to absorb reflected waves, and prevent them from falsely interfering with the incoming light. At the bottom, the cell layers are stacked successively on top of each others with the bow-tie on top of them. This later structure excites surface plasmon waves on the metal layer surface^35^. This excitation happens when a plane wave having its magnetic vector along the $y-$axis—the long axis of the bow-tie—reaches the nanostructure under normal incidence conditions. In this case, the surface plasmon waves will propagates along the $x-$axis direction. We select the amplitude of the magnetic field as $H_{y0}= 1$ A/m, being the magnetic field vector $\mathbf {H} = (0, H_{y0}e^{-ik_z.z}, 0)$. The light source emits at $\lambda = 632.8$ nm that illuminates the device with an irradiance of 50 mW/cm$^2$. This value is achievable using regular diode lasers. Actually, we can select any monochromatic optical source with a wavelength lying within the absorption band of the device that is much wider than the spectral width of most of laser diodes^36,37^. Then, we can optimize the period of the design to work for the selected wavelength.

In computational electromagnetism, the selection of an appropriate meshing is a key factor to assure reliable and robust results. In our case, physical domains use tetrahedral elements while perfect matching layer (PMLs) use a prismatic mesh. The density of the meshing elements is related to the dimension of the smallest structure, and should be equal or less than $\lambda /5$^38,39^. A finer mesh leads to better convergence of the solution at a cost of increasing computational resources. Having these criteria into account and considering that our unit cell does not contain extreme tiny structures, we have selected a minimum mesh size around $\lambda /10$ for the region close to the interfaces, and $\lambda /7$ for larger domains. The average number of mesh elements reaches $10^5$.

### Design and parameterization

**Figure 1:**
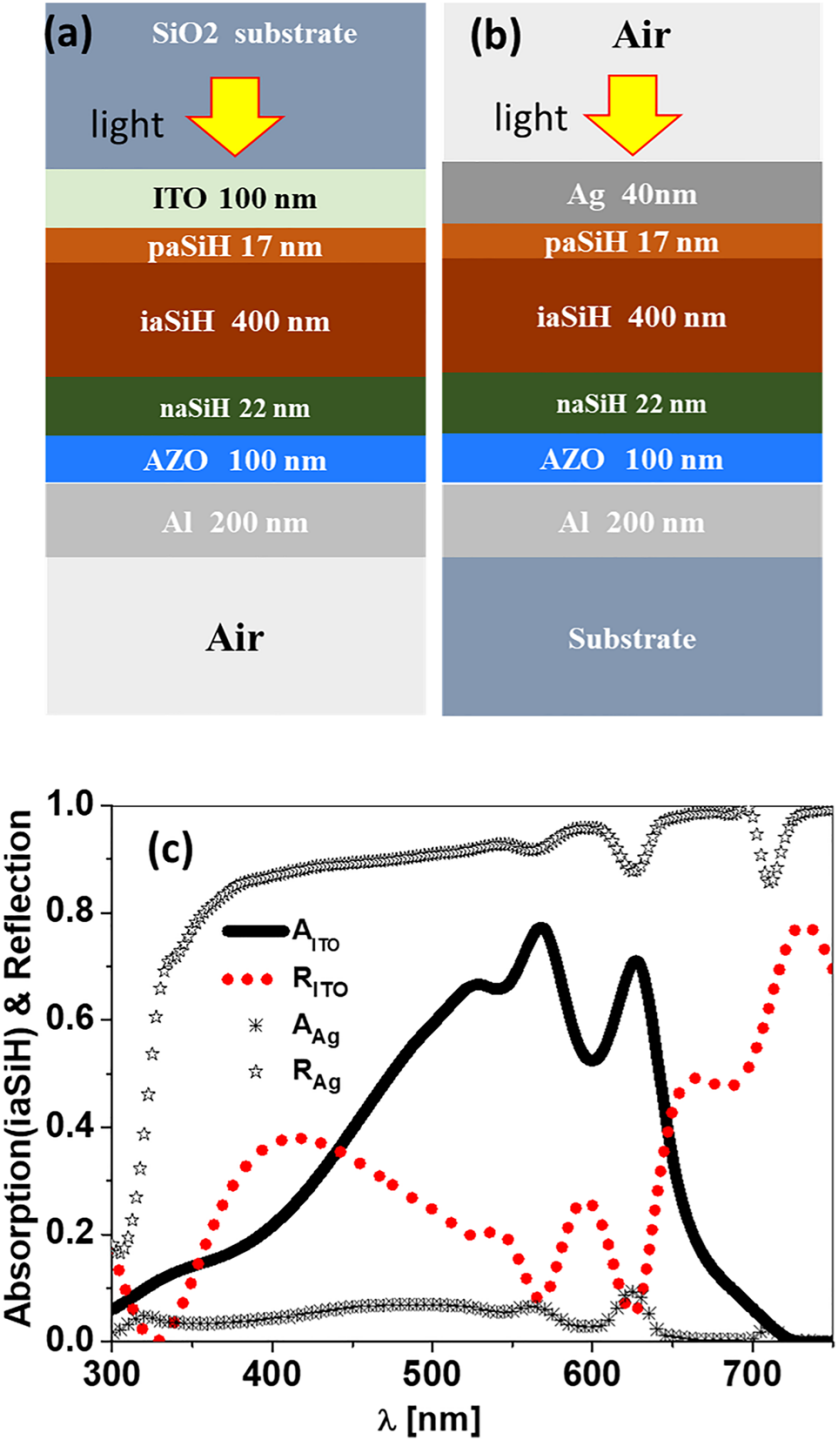
(**a**) Layered structure of conventional aSiH thin film solar cell with top transparent electrode made of ITO. (**b**) The same device where ITO is replaced with a non transparent metallic contact from Ag. (**c**) Absorption at the active layer and total reflectance of a conventional aSiH thin film solar cell with ITO electrode ($A_\mathrm{ITO}$, $R_\mathrm{ITO}$) and for the device with Ag electrode ($A_\mathrm{Ag}$, $R_\mathrm{Ag}$).

The layered structure of a conventional aSiH thin film solar cell is presented in Fig. 1a. As light incides from the substrate (from top), it finds the following materials and layers: SiO$_2$ glass substrate—indium tin oxide, ITO, transparent electrode (100 nm-thick)—ptype aSiH (paSiH) buffer layer (17 nm-thick)—intrinsic aSiH (iaSiH) active layer (400 nm-thick)—ntype aSiH (naSiH) buffer layer (22 nm-thick)—aluminum doped zinc oxide, AZO (100 nm-thick)—aluminum reflecting electrode (200 nm-thick)^33,40^. This device has a broadband absorption at the iaSiH active layer in the spectral range (300–720) nm, combined with a total low reflectance due to the transparency of the ITO electrode.

In our design, we replace the top transparent electrode, ITO by a 40 nm-thick silver electrode (see Fig. 1b). Silver is selected because of its narrow plasmonic response. However, if robustness against environmental agents is an issue, as it could happens with liquid analytes, we can passivize this layer by adding a very thin gold layer that do not affect the overall behavior of the system^41^. The response of the device with layer structure Fig. 1a is shown in Fig. 1c, where we represent the absorption of the active layer, that will be related with the short circuit current delivered by the cell, $A_\mathrm{ITO}$, and the total reflection of the cell $R_\mathrm{ITO}$, where the $\mathrm{ITO}$ subscript refers to the presence of the transparent electrode. These characteristics are key when customized the device to work as an efficient solar energy harvester based on the photovoltaic conversion^33^. However, for the device in Fig. 1b because of this electrode replacement, the absorption at the active layer is strongly reduced, and most of the incoming light is reflected back (see $A_\mathrm{Ag}$, and $R_\mathrm{Ag}$ in Fig. 1c). The Ag layer precludes the use of the device as an energy harvesting element and prepares the structure for the next step where SPRs generate the desired selective response. The thickness of the Ag layer is set at 40 nm to facilitate the transmission of the SPR to the active layer. A thinner layer would be not selective enough, and a thicker one would strongly block the light reaching the active layer. This value is within the range previously reported in the literature for devices based on the generation of SPRs^42–44^. From a fabrication point of view, the design is flipped, being the Al back electrode directly deposited on the substrate (see Fig. 1b) meanwhile the incidence is towards the metallic silver electrode. The response of the flipped device is independent of the substrate material, allowing the use of flexible, low-cost, plastic substrates. This design strongly blocks the direct transmission of the light to the active layer, delivering a quite low photo-electric response. The next step is to selectively activate the absorption by exciting a SPR. Our proposal incorporates a periodic array of bow-tie structures that scatters the light and excites plasmonic resonances with a narrow line-shape. The optical field within the active layer greatly enhanced at the resonance wavelength of the SPR mode. So, the narrow spectral response characteristics of the SPR is translated to the response of the cell. Although many nano objects can be applied for this purpose, we have chosen a dielectric bow-tie resonant structure because it has a wide band scattering capabilities^45^. This generates a multimode plasmonic response when placed close to a thin metal film. Besides, choosing a transparent dielectric material for the bow-tie reduces the reflection and absorption losses of the object itself.

**Figure 2:**
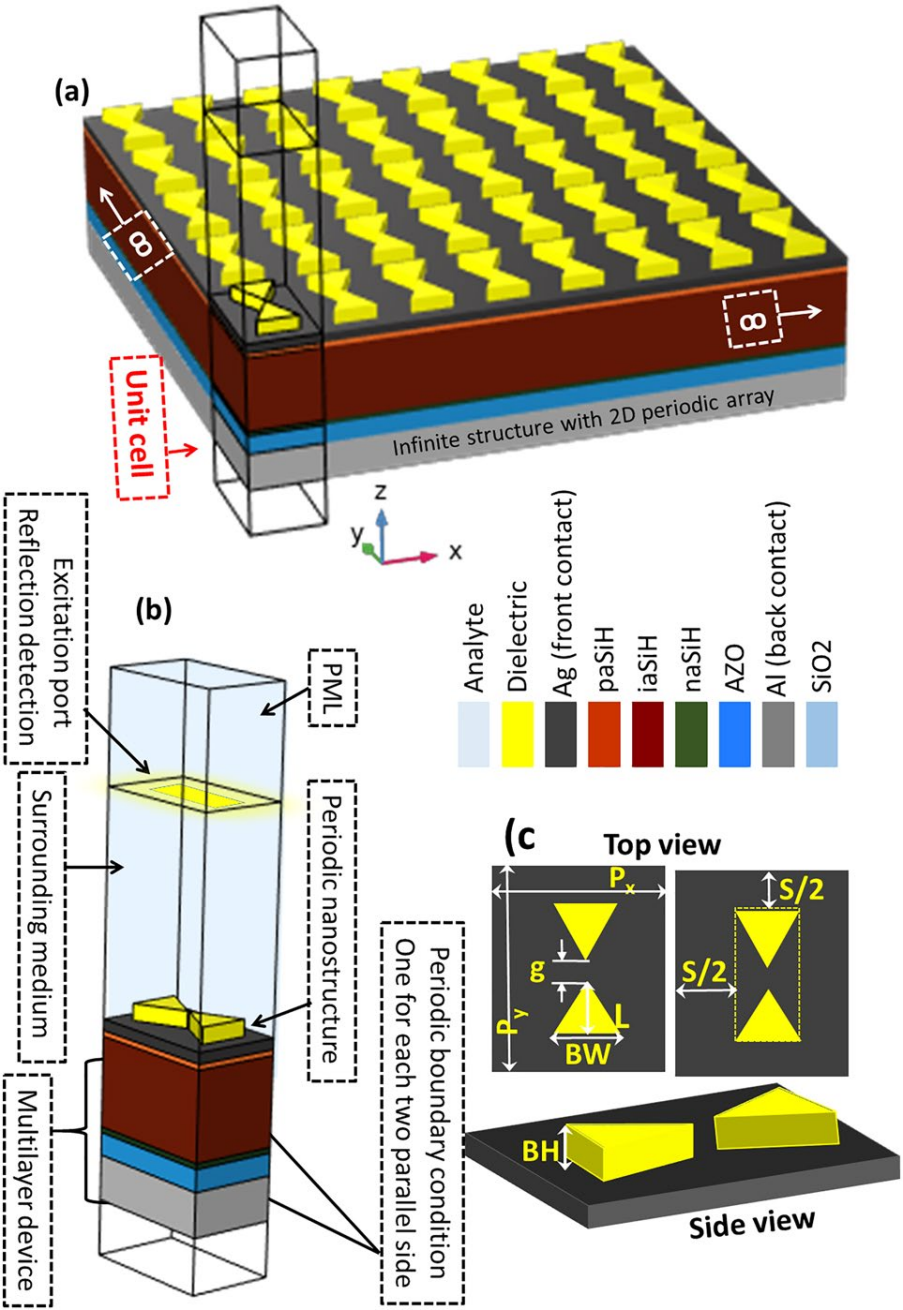
(**a**) 3D representation of the proposed device with an infinite array of bow-tie resonant structures. The unit cell is highlighted on the left bottom corner. (**b**) Detailed arrangement of the unit cell including the excitation port, the boundary condition represented as periodic conditions at the lateral surfaces and as a perfect matche layer (PML) on top, the multilayer structure of the device, the top analyte medium, and the substrate. The color index on the middle defines all the materials included in the device. (**c**) Top and side view of the proposed structure with all the geometrical parameters.

A 3D representation of the proposed design is shown in Fig. 2a. The unit cell, marked at the left-bottom corner of the plot, and detailed in Fig. 2b. At the bottom, the cell layers stacked successively on top of each others with the bow-tie on top of them.

The top (analyte) and bottom (substrate) domains are defined as semi-infinite. In Fig. 2c, the geometrical parameters of the bow-tie unit cell are depicted. The bow-tie antenna has two triangular shape elements of base width *BW* and length *L*, separated by a gap, *g*, so the total length of the bowtie is $L_t=2L+g$. The dielectric structure is centered in a rectangle having the following size

1 $$\begin{aligned} P_x= BW+S ,\end{aligned}$$

2 $$\begin{aligned} P_y= L_t+S ,\end{aligned}$$

where *S* is the separation between bow-ties along each direction (we consider the same separation along $x-$ and $y-$axis), and $P_x$ and $P_y$ are the spatial periods of the infinite arrangement of the unit cell. An additional geometrical parameter of the unit cell is the thickness of the dielectric bow-tie, *BH*. In Table 1, we summarize the geometry and material selection of the layers of the proposed structure. Each layer of the device is defined using its wavelength dependent complex refractive index data obtained from recognized sources as indicated in Table 1.

**Table 1:** Geometric and material arrangement of the device.

Layer	Shape and thickness	References
analyte (initially vacuum n$_a$=1.0)	Infinite	
bowtie	base width *BW*, height *BH*, total length $L_t=2L+g$, periods $P_x,P_y$	
Ag	Thin film, 40 nm	^46^
paSiH	Thin film, 17 nm	^40^
iaSiH	Thin film, 400 nm	^40^
naSiH	Thin film, 22 nm	^40^
AZO	Thin film, 100 nm	^40^
Al	Thin film, 200 nm	^46^
SiO$_2$	Infinite substrate	

## Results and discussion

Every optimization begins with the definition of a meaningful merit function that best describes the desired performance of the device. In our case, we want to maximize the short circuit current delivered by the detector, $J_\mathrm{sc}$. This value is proportional to the absorption at the active layer of the device. Therefore, our merit function is this absorption $A_\mathrm{iaSiH}$, that was already evaluated in Fig. 1c. We will make an evaluation of the absorption in the active layer of the cell in terms of the geometrical parameters of the bow-tie *BW*, and *S* for different bow-tie materials.

For our first optimization step we set the height of the bow-tie to $BH=150$ nm, and the gap between the two triangular portions to $g=20$ nm. For the sake of simplicity the base and the length of the triangular portions of the bowtie are equal, $BW=L$. Then, we only work with *S* and *BW* as free parameters to present two-dimensional maps of the merit function. This analysis is made for three dielectric materials of the bow-tie. These materials are MgF$_2$ ($n_a \approx$ 1.37), SiO$_2$ (($n_a \approx$ 1.47), and AIN ( $n_a \approx$ 2.1). These three materials correspond to low, medium, and high index of refraction at $\lambda =632.8$ nm, respectively.

**Figure 3:**
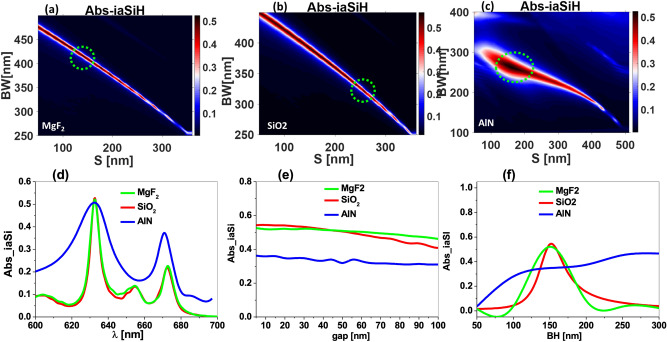
Absorption in the active layer as a function of the *BW* and *S* parameters of the bow-tie. The materials of the bow-tie are (**a**) MgF$$_2 $$, (**b**) SiO$$_2$$, and (**c**) AlN. The dashed circles represent the location of the maximum absorption at the active layer of the cell. (**d**) Spectral absorption at the active layer for the selected optimized parameters for each material. The role of the gap separation between the triangular portions of the bow-tie, *g*, is shown in (**e**), and the dependence with respect to the thickness of the dielectric structure, *BH*, is plotted in (**f**), for the three dielectric materials considered in this analysis. All these calculations have been made at $$\lambda =632.8$$ nm (except for subplot **d**).

The size of the dielectric bow-tie *BW* tunes its spectral scattering, meanwhile the separation, *S*, determines the strength of the coupling between adjacent bow-ties. Both effects have a direct impact on the spectral location and shape of the SPRs excited by the structure. Figure 3a–c are maps of the merit function in terms of *BW* and *S* for the three dielectric materials under analysis (MgF$_2$, SiO$_2$, and AIN). We can see that a higher absorption is obtained for low values of *S*, which means closer bow-ties. From the plots, we can define an optimum separations of around $S=145, 260, 180$ nm for MgF$_2$, SiO$_2$, and AIN, respectively. The comparative analysis of these maps allows to conclude that the maximum absorption at the active layer appears for smaller values of the *BW* of the bow-tie as the index of refraction of the bow-tie increases. We find this maximum absorption at $BW= 415, 320, 260$ nm for MgF$_2$, SiO$_2$, and AIN, respectively.

A quite interesting feature is that the maximum value of the absorption is about the same for the three materials considered in this analysis, being SiO$_2$ the one showing the largest value. To compare between the responses of device for all materials, we have calculated the spectral absorption (see Fig. 3d). This analysis show how the linewidth is narrower for SiO$_2$ and MgF$_2$. However, the contrast between the index of refraction of the analyte (n$_a$ near 1) and the scatterer is larger for SiO$_2$ than for MgF$_2$. This fact will be important when considering the signal change obtained when the index of refraction of the analyte varies^47^.

After optimizing the absorption at the active layer with an appropriate choice of the geometry (considering *BW* and *S*) and materials, we have analyzed the role of the remaining geometric parameters. When the gap separation between the portions of the dielectric bow-tie, *g*, increases, we observe a slight, but consistent, decrease in the absorption at the active layer (see 3e), especially for *g* larger than 40 nm. On the other hand, this absorption shows a maximum for a value of $BH=150$ nm (see Fig. 3f), allowing a reasonable departure from this value ($\pm 10$ nm) without a significant reduction of absorption. Therefore, for the SiO$_2$ structures, the selected values for these two parameters are: $g=20$ nm, and $BH=150$ nm.

**Figure 4:**
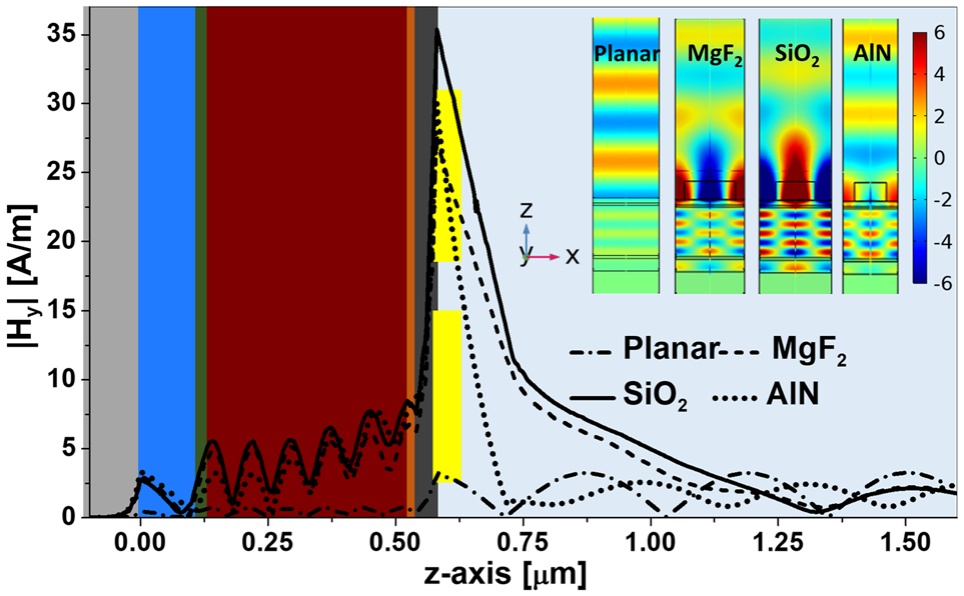
A line plot for the magnetic field y-component through the whole device without the bow-tie (black line) and with the bow-tie made of MgF$$_2$$ (green line), SiO$$_2$$(red line), and AlN (blue line). The magnetic field inside the active layer is greatly enhanced due to the excitation of SPRs when including the bow-ties. The inset is for the magnetic field distribution y-component $$H_y $$ in the *xy* plane at $$ \lambda = 632.8$$ nm for the planar structure (no resonant elements) and the case of the bow-ties fabricated with the three selected materials.

To better understand the electromagnetic behavior of the device, we have plotted the modulus of the magnetic field along the *z*-axis passing through all layers of the device at the resonance wavelength, $\lambda$=632.8 nm. Figure 4 shows this line for the device with the bow-tie made of MgF$_2$ (green line), SiO$_2$ (red line), AlN (blue line), and for the device without bow-tie (black line). The magnetic field reaches its maximum value at the region close to the metal/dielectric interface, where the SPRs are excited using the bow-tie. The magnetic field in the active layer and at the interface is greatly large for the device with bow-ties compared to the device without bow-tie. The penetration depth of the field at the analyte medium on top of the cell is largest for SiO$_2$, and lowest for AlN. This means a higher interaction volume for the SPR generated by the SiO$_2$ bow-tie, and hence more sensitivity to variations of the analyte. This effect enhances the available optical power at the active layer, generating a larger photovoltaic response. The inset of Fig. 4 is for the spatial distribution of the magnetic field at the *xz* plane for the device using each bow-tie material, showing the SPR characteristic decaying maxima, and the corresponding field in the active layer. The case of the planar cell structure, without resonant elements is also plotted in the figure, showing how the field is enhanced when including the bow-tie arrangement.

### Performance as a refractometric sensor for gases

The sensing performance of a refractometric sensor is evaluated through the sensitivity, $S_B$, and the FOM^18^ for the geometrical parameters and material choice already discussed previously at wavelength of $\lambda =632.8$ nm, which is a constant in our design.

Our design is interrogated electronically, then its responsivity, $\mathcal{R}$, is defined as the quotient between the current extracted from the device, $I_\mathrm{signal}$ , and the incident optical power $P_\mathrm{input}$. As expected for a plasmonic sensor, $\mathcal{R}$ is a function of the refractive index of the analyte.

The sensitivity of this sensor can be obtained form the variation of $\mathcal{R}_{S}$ with respect to the index of refraction of the analyte, $n_a$^48,49^.

3 $$\begin{aligned} S_B = \frac{\partial \mathcal{R}_{S}}{\partial n_a} , \end{aligned}$$

and the FOM is also redefined in terms of the responsivity, $\mathcal{R}_{S}$^48,49^ as:

4 $$\begin{aligned} \mathrm{FOM} = \frac{S_B}{\mathcal{R}_{S}} . \end{aligned}$$

A gas under atmospheric pressure shows a very little change in the index of refraction (with an order of magnitude of 10$^{-4}$) when physical or chemical parameters of the atmosphere under test vary. For example, the difference between the indexes of refraction of vacuum, $n_\mathrm{vacuum}=1$, and air is in the fourth decimal digit: $n_\mathrm{air}=1.000298$. This value is related with their relative content of nitrogen ($n_{\mathrm{N}_2}=1.000297$) and oxygen ($n_{\mathrm{O}_2}=1.000272$)^50^. The detection of those tiny variations requires high-resolution devices^50^.

**Figure 5:**
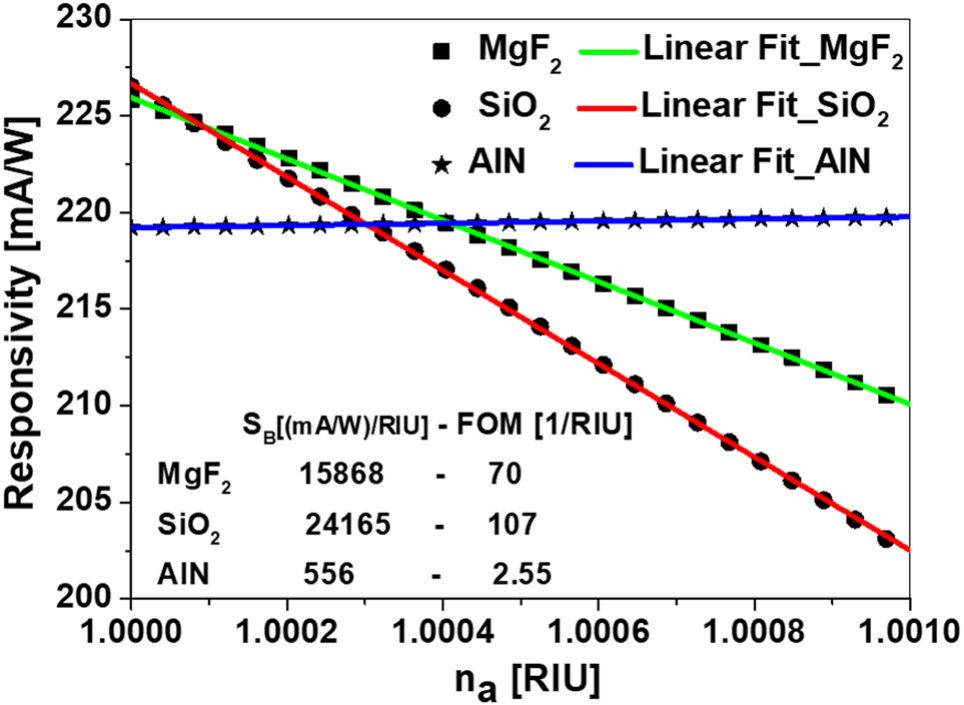
Responsivity, $\mathcal{R}$, as a function of the refractive index of the analyte, $n_a$, for the device with the optimized geometry of the bow-tie made of MgF$_2$ (black squares), SiO$_2$ (black circles), and AlN (black stars). The linear fitting of these simulated data are ploted in green for MgF$_2$, red for SiO$_2$, and line for AlN. The values of $S_B$, and FOM are listed within the plot. The device is illuminated with a monochromatic light source, $\lambda =632.8$ nm.

For our device, the analyte is in contact with the dielectric bow-tie and the front metal layer. The electric signal delivered by the system is generated by the absorbed optical power at the active layer, that is enhanced through the excitation of SPR. The dependence of the plasmonic resonance with the index of refraction of the analyte provides the desired functionality of the device. The device is highly sensitive to the presence of gas contentment in air. for example, using the device with bow-tie made of SiO$_2$, the responsivity of the device drops from 219 mA/W for air to 215.6 mA/W if air is replaced with CO$_2$. At this point, we should remind that refractometric sensors only respond to changes in the index of refraction of the environment, disregarding the actual origin of this change. Then, the quantitative use of our design should be limited to the case of closed atmospheres, where the gas species are known and not changed. The responsivity values of the device with a bow-tie made of each material as a function of the analyte refractive index, $n_a$ are presented in Fig. 5. The $S_B$ values extracted from the slope of the linear fitting for each data set, and revealed as 1.58x10$^4$(mA/W)/RIU, 2.4x10$^4$ (mA/W)/RIU, and 558 (mA/W)/RIU, for the device with a bwo-tie made of MgF$_2$, SiO$_2$, and AlN, respectively. The corresponding FOM for each case are obtianed by dividing the $S_B$ on the maximum responsivity for each data set, which reveals a 70 1/RIU, 107 1/RIU, and 2.55 1/RIU for the device with a bwo-tie made of MgF$_2$, SiO$_2$, and AlN, respectively.

## Conclusions

In this contribution we have taken advantage of the maturity of aSiH photovoltaic solar cells. The addition of a customized metasurface made of an array of dielectric bow-ties on top of a metallic layer, has transformed the energy harvester device into a refractometric sensor. In this transformation, we have also replaced the transparent electrode by a thin-film metallic one. This metal layer is thick enough to excite plasmon resonances that are strongly dependent on the index of refraction of the analyte. The main advantage of this approach is that the change of the index of refraction of the analyte is translated to the response of the device, allowing a simpler design and operation. Then, the sensor becomes opto-electronically interrogated. The optical absorption is maximized by the geometrical parameters of the bow-tie arrangement through the excitation of the SPR. Therefore, we have analyzed in detail how the geometry can be tailored to enhance absorption at the active layer and generate a quite selective response in wavelength and index of refraction related with the conditions for the excitation of SPR. The device can be operated by exposing the bow-tie array and the metallic front thin-film to the atmosphere. As a sensor, its performance is quantitatively given by the value of its sensitivity of $S_B= 2.4 \times 10^4$ (mA/W)/RIU, and $\text{ FOM } = 107$ (1/RIU) for the case of bow-ties made of SiO$_2$.

As a summary of this contribution, our proposed design opens the way for plasmonic refractometric sensor to be incorporated in simpler and more compact designs, just by exploiting the advantages of photovoltaic technologies and metasurfaces.

## Data Availability

The datasets used and analysed during the current study are available from the corresponding author on reasonable request.
