# Author Correction: Optoelectronic refractometric sensing device for gases based on dielectric bow‑ties and amorphous silicon solar cells

**DOI:** 10.1038/s41598-023-29200-z

**Published:** 2023-02-02

**Authors:** Mahmoud H. Elshorbagy, Óscar Esteban, Alexander Cuadrado, Javier Alda

**Affiliations:** 1 Physics Department, Faculty of Science, Minia University, 61519 El‑Minya, Egypt; 2 Faculty of Optics and Optometry, Applied Optics Complutense Group, University Complutense of Madrid, C/Arcos de Jalón, 118, 28037 Madrid, Spain; 3 Photonics Engineering Group, University of Alcalá, 28801 Alcalá de Henares, Madrid, Spain; 4 Escuela de Ciencias Experimentales y Tecnología, University Rey Juan Carlos, 28933 Móstoles, Madrid, Spain

Correction to: *Scientific Reports* https://doi.org/10.1038/s41598-022-21299-w, published online 01 November 2022

The Acknowledgements section in the original version of this Article was incomplete.

“This work has been partially supported by the Spanish Ministry of Science, Innovation and Universities projects NERA under Grant RTI2018-101037-B-I00, and NANOROOMS under Grant PID2019-105918GB-I00, by AEI/FEDER funds and by Comunidad de Madrid and FEDER Program SINFOTON2-CM under Grant S2018/NMT-4326, and TELURO under Grant RTC2019-007113-3.”

now reads:

“This work has been partially supported by the Spanish Ministry of Science, Innovation and Universities projects NERA under Grant RTI2018-101037-B-I00, and NANOROOMS under Grant PID2019-105918GB-I00, by AEI/FEDER funds and by Comunidad de Madrid and FEDER Program SINFOTON2-CM under Grant S2018/NMT-4326, and TELURO under Grant RTC2019-007113-3. This work was partially supported by Proyectos de I+D para jóvenes investigadores de la Universidad Rey Juan Carlos financiado por la Comunidad de Madrid, Codigo 2022/00156/025, REF:M2742.”

The original Article has been corrected.

